# Supplementary figures and images for: Three New Integration Vectors and Fluorescent Proteins for Use in the Opportunistic Human Pathogen Streptococcus pneumoniae
Source: Genes (Basel). 2019 May 22;10(5):394. doi: 10.3390/genes10050394 (PMC6562690; doi:10.3390/genes10050394)

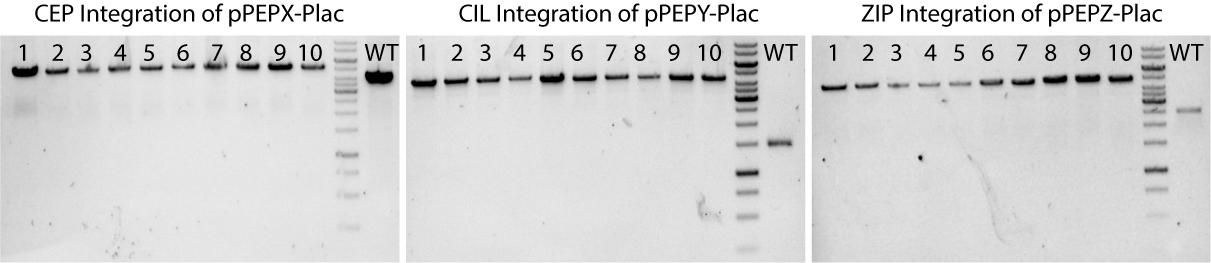

Supplement: Supplementary file 1 [file genes-10-00394-s001.zip › Supplementary Figures/sup figure 1.tif]

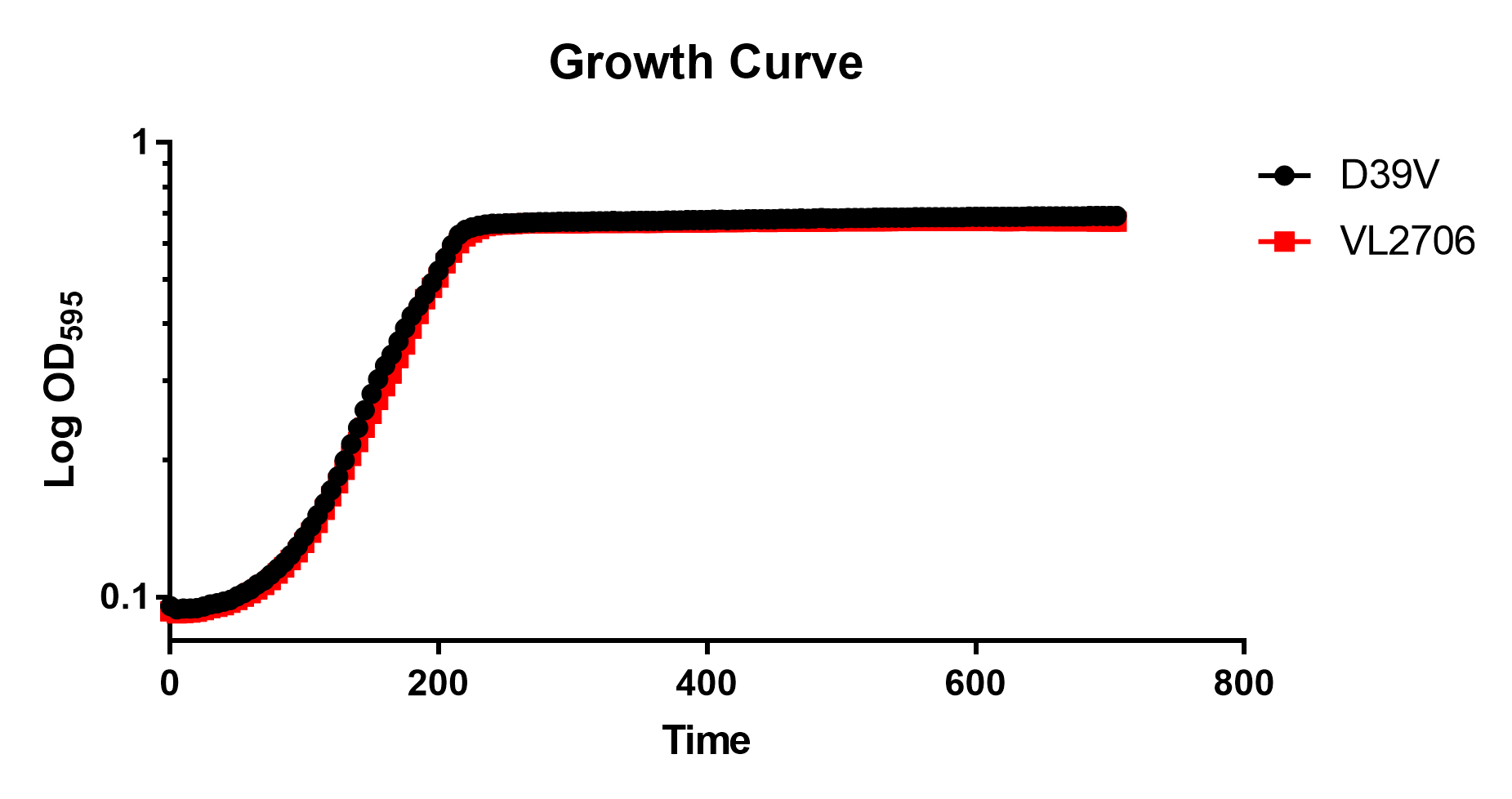

Supplement: Supplementary file 1 [file genes-10-00394-s001.zip › Supplementary Figures/sup figure 2.tif]

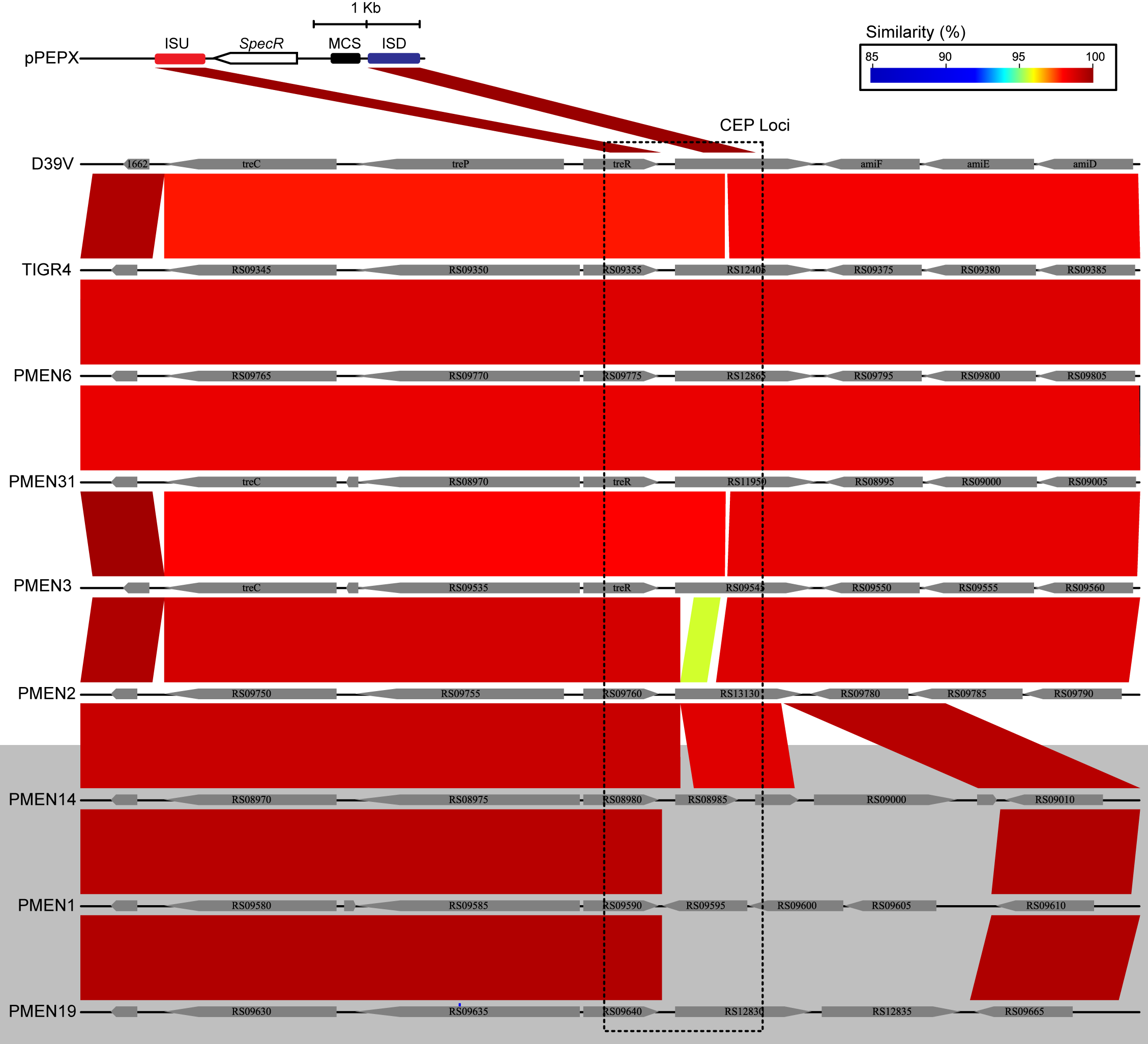

Supplement: Supplementary file 1 [file genes-10-00394-s001.zip › Supplementary Figures/sup figure 3.tif]

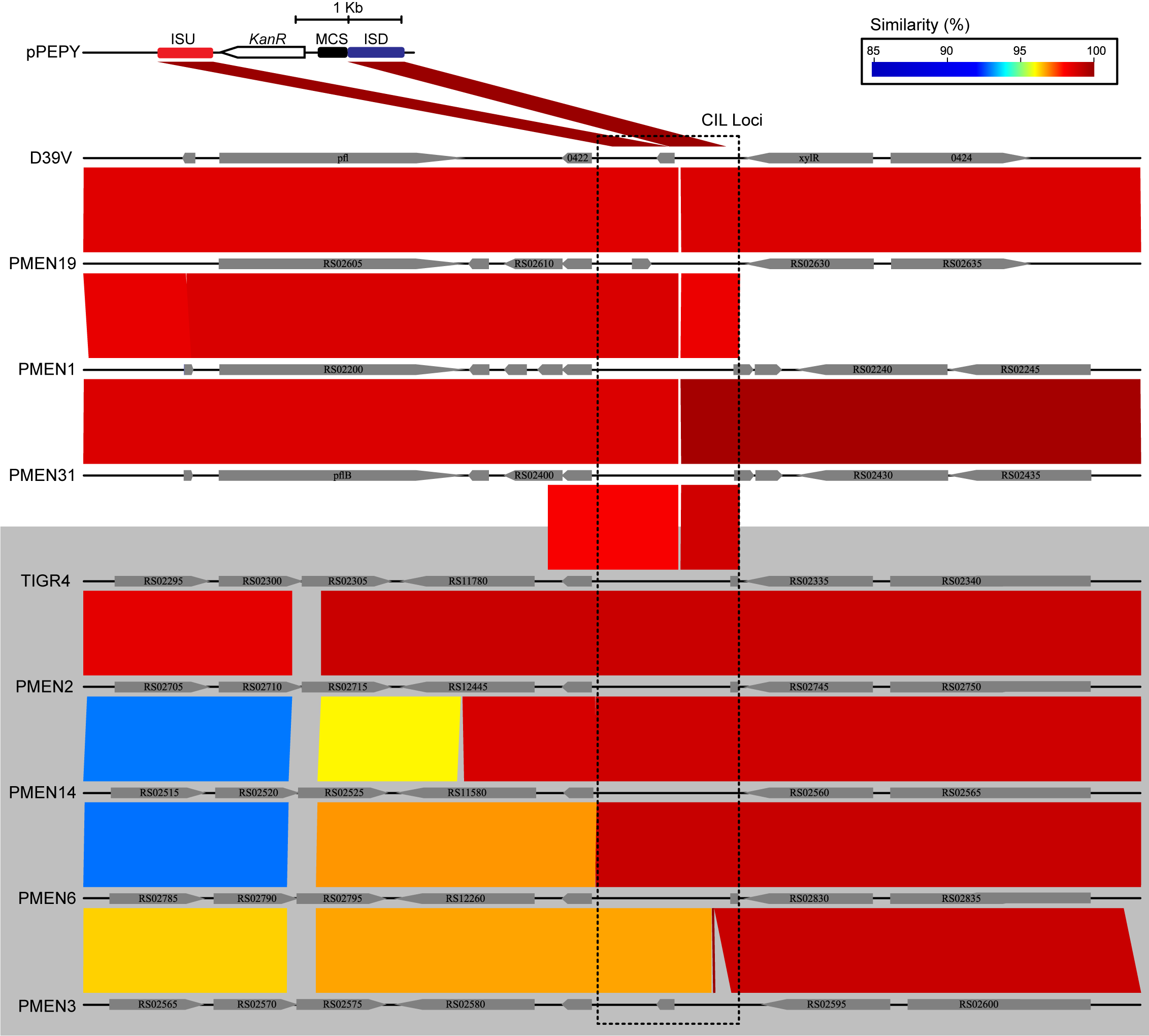

Supplement: Supplementary file 1 [file genes-10-00394-s001.zip › Supplementary Figures/sup figure 4.tif]

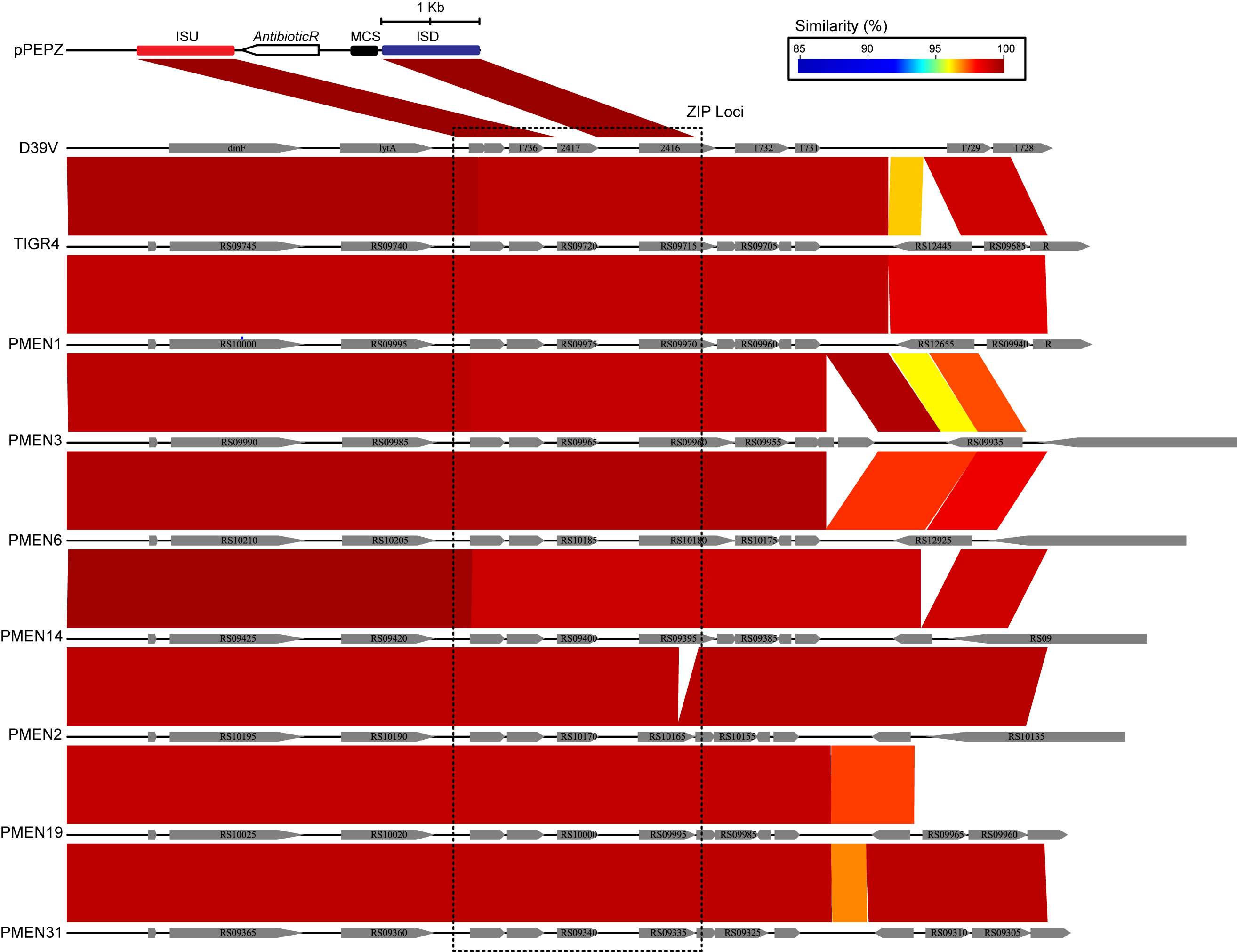

Supplement: Supplementary file 1 [file genes-10-00394-s001.zip › Supplementary Figures/sup figure 5.tif]
